# In Vitro Assessment of Lyophilized Advanced Platelet-Rich Fibrin from Dogs in Promotion of Growth Factor Release and Wound Healing

**DOI:** 10.3390/vetsci9100566

**Published:** 2022-10-15

**Authors:** Ravisa Warin, Preeyanat Vongchan, Witaya Suriyasathaporn, Ratchadaporn Boripun, Wanna Suriyasathaporn

**Affiliations:** 1 Graduate Program in Veterinary Science, Faculty of Veterinary Medicine, Chiang Mai University, Chiang Mai 50100, Thailand; 2 Department of Medical Technology, Faculty of Associated Medical Sciences, Chiang Mai University, Chiang Mai 50200, Thailand; 3 Department of Food Animal Clinic, Faculty of Veterinary Medicine, Chiang Mai University, Chiang Mai 50100, Thailand; 4 Research Center of Producing and Development of Products and Innovations for Animal Health and Production, Chiang Mai University, Chiang Mai 50100, Thailand; 5 Nagoya University Asian Satellite Campuses Institute-Cambodian Campus, Royal University of Agriculture, Dangkor District, Phnom Penh 370, Cambodia; 6 Akkhraratchakumari Veterinary College, Walailak University, Nakhon Si Thammarat 80160, Thailand; 7 Department of Companion Animals and Wildlife Clinic, Faculty of Veterinary Medicine, Chiang Mai University, Chiang Mai 50100, Thailand; 8 Center of Elephant and Wildlife Health, Chiang Mai University, Chiang Mai 50100, Thailand

**Keywords:** lyophilization, advanced platelet-rich fibrin, growth factors, wound healing, tissue regeneration, regenerative medicine, dogs

## Abstract

**Simple Summary:**

Advanced platelet-rich fibrin (A-PRF) induces more migration and proliferation of fibroblasts compared with standard PRF, but it being freshly prepared prior to it being applied is necessary. To preserve its biological function, lyophilization, a freeze-drying method, has been developed to improve the stability and storage potential of PRF. This study aimed to determine the effect of lyophilized A-PRF on growth factor release and cell biological activity compared to fresh A-PRF. The results of the present study demonstrated that both formulations of canine A-PRF matrices, the fresh and lyophilized forms, were able to release various growth factors, promoting better wound regeneration than in instances without the A-PRF matrices. Lyophilization in canine A-PRF can mostly preserve the similar release of growth factors, and consequently has similar biological activities to a fresh preparation. Interestingly, lyophilized canine A-PRF demonstrated the tendency of larger releases of certain growth factors, including the significantly larger accumulated release of a growth factor named vascular endothelial growth factor-A (VEGFA). Based on these findings, it became clear that the lyophilization process can preserve growth factor release as well as the biological activity of canine A-PRF matrices, in addition to encouraging the use of canine lyophilized A-PRF as a biological material for promoting wound healing.

**Abstract:**

Advanced platelet-rich fibrin (A-PRF) induces more proliferation and migration of fibroblasts compared with standard PRF, but it being freshly prepared prior to it being applied is necessary. Therefore, this study aimed to determine the effect of lyophilized A-PRF on growth factor release and cell biological activity. Blood samples were collected from six dogs and processed for fresh and lyophilized A-PRF. The growth factors released included transforming growth factor beta-1 (TGF-β1), vascular endothelial growth factor-A (VEGFA), and platelet-derived growth factor-BB (PDGF-BB), and the fibroblast proliferation as well as wound closure enhancement of both products were compared. The results showed that TGF-β1, PDGF-BB, and VEGFA were continually released from lyophilized A-PRF for over 72 h. Lyophilized A-PRF released significantly more accumulated VEGEA and a tendency to release more TGF-β1 at 72 h as well as VEGFA at 24 h and 72 h than fresh A-PRF. Moreover, lyophilized A-PRF increased fibroblast proliferation and induced a significantly faster wound closure than the control, while no significant difference between fresh and lyophilized A-PRF was found. In conclusion, the lyophilization of canine A-PRF can preserve the release of growth factors and has similar biological activities to a fresh preparation. This encourages the substitution of lyophilized A-PRF instead of fresh A-PRF in regenerative treatments in which the stability of the product is concerned.

## 1. Introduction

Wound management expenditures in domestic and livestock animals cost approximately USD 1.1 billion in 2021, and are predicted to see a 7% increase per year [1]. A chronic wound is a wound without complete healing in a normal period despite appropriate bandaging and treatments [2]; consequently, it entails long-term medical treatment and healthcare costs [3]. Apart from normal wound healing processes, involving hemostasis, inflammation, proliferation, and remodeling [4], chronic wounds cause excessive inflammation, persistent infection, and the impaired response of epithelial cells to reparative stimuli [5]. To accelerate the healing process in chronic wounds, a variety of therapeutic materials have been developed including nanomedicine [6], hydrogel [7], natural polysaccharides, and phenolic compounds and proteins [8] in regenerative medicine technology. However, platelets for wound care improvement have been proposed to be a reliable technique [9] with probably the lowest price. Platelets play an important role in wound healing processes, including facilitating fibrin clot formation, enhancing angiogenesis, and promoting granulation tissue formation by releasing growth factors, such as platelet-derived growth factors (PDGFs), vascular endothelial growth factors (VEGFs), and transforming growth factors (TGFs) from platelet alpha granules [10]. 

Platelet-rich plasma (PRP) is the first generation of concentrated platelets, and has been widely utilized for promoting wound repair for many years [11]. Despite its widespread use, in recent years some studies have suggested that the anticoagulation factor in PRP can delay wound healing [12]. Moreover, almost all PRP preparations are complicated and expensive [13]. With this limitation, the development of platelet-rich fibrin (PRF) was initiated by Choukroun and colleagues [14,15] to provide a provisional matrix for cell recruitment, tissue regeneration, and prolonged growth factor release [16,17,18]. Initially, PRF has been studied in human medicine, especially dentistry; for instance, PRF increased in vitro gingival fibroblast proliferation [19,20] on extraction sockets, gingival recession, and palatal wound closure [21]. In 2014, a new PRF preparation, so-called advanced PRF (A-PRF), was developed [22] by decreasing the centrifugation force (g) to 100 g for 14 min, and A-PRF produced more growth factors [23], proliferation, and cell migration of gingival fibroblasts [24]. Both in vitro and in vivo research have supported PRF as a biomaterial strategy for the treatment of a wide variety of types of wounds in humans and animals [9,25,26]. Recently, information on the application of canine PRF to wound healing has been encouraged for veterinary use [27,28]. However, the application of PRF remains reliant upon fresh preparations due to the short half-life of the product [29].

To preserve the biological function, lyophilization, a freeze-drying method, has been developed to improve the stability and storage potential [30,31]. In the human context, lyophilized PRF has been proven to promote craniofacial bone regeneration [32]. In soft tissue wound healing, a dressing composed of polyvinyl alcohol hydrogel containing granule-lyophilized PRF could accelerate wound healing in acute full-thickness skin wounds in a mouse model [33]. However, no study is available on the effect of lyophilization for A-PRF, an advanced technique of producing PRF, on the release of growth factors related to wound healing processes, especially in veterinary medicine. Therefore, this study aimed to determine the efficacy of fresh A-PRF and lyophilized A-PRF in promoting the wound healing process in vitro using canine blood samples. Growth factor release and wound healing properties, including the proliferation of fibroblasts and wound closure, were evaluated.

## 2. Materials and Methods

### 2.1. Ethical Approval, Animal Selection, and Study Design 

Animal use protocols were approved and permitted by the Animal Care and Use Committee, Faculty of Veterinary Medicine, Chiang Mai University, Thailand (FVM-CMU-ICUC Ref. No. S27/2563). The experimental protocols were accompanied by laboratory animal ethics laws, and all efforts were made to decrease animal suffering and the number of animals used. Dog owners were informed and gave their consent before participation.

Six clinically healthy Labrador Retriever dogs (three males and three females) from Border Patrol Police Division 33, Chiang Mai, Thailand, were evaluated for their health statuses and had no concurrent diseases; any history of antiplatelet or anticoagulant medication use was included. The health statuses were evaluated via a physical examination and blood screening, including hematology, blood biochemistry, and a blood smear for a blood parasite examination. Their mean ± sd weight was 32.8 ± 5.9 kg, with a range from 24 to 40 kg. Their mean ± sd age was 4.1 ± 1.1 years, with a range from 2 to 5 years. Each dog was raised in a separate kennel under standard environmental conditions (29–32 °C, 55 ± 5% humidity, and a 12 h light/dark cycle), with permission to be free-range twice daily. They were given free access to water and certified commercial pellet food once daily. 

**Figure 1:**
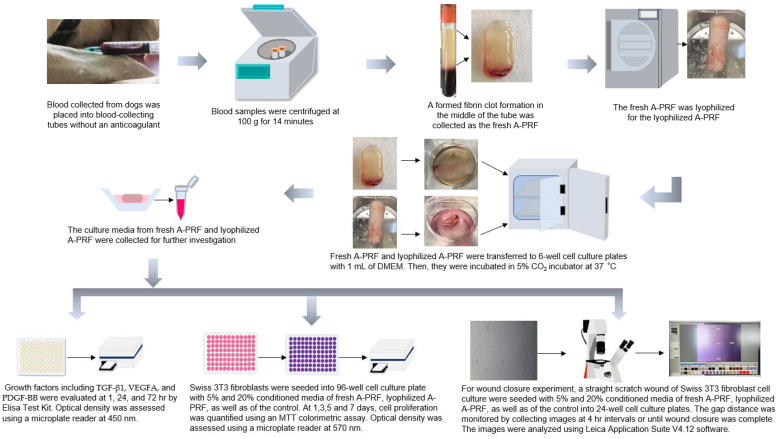
Schematic outline of experiments comparing the fresh canine advanced platelet-rich fibrin (fresh A-PRF) and the lyophilized canine advanced platelet-rich fibrin (lyophilized A-PRF) on growth factor release, including transforming growth factor beta-1 (TGF-β1), vascular endothelial growth factor-A (VEGFA), and platelet-derived growth factor-BB (PDGF-BB), in addition to the enhancement of fibroblast proliferation and wound closure. DMEM: Dulbecco’s Modified Eagle Medium.

For the study design, briefly, two samples of 10 mL of blood from each dog were collected and processed for the fresh A-PRF and lyophilized A-PRF, and subsequently their releases of growth factors, including TGF beta-1 (TGF-β1), VEGF-A (VEGFA), and PDGF-BB (PDGF-BB), were evaluated at 1, 24, and 72 h. For determining wound healing properties, the fibroblast cells were cultured with a control, fresh A-PRF, and lyophilized A-PRF. Cell proliferation was quantified by an MTT assay at 1, 3, 5, and 7 days. Wound closure was evaluated by a scratch wound assay, in which the gap distance of the scratch on the cell was measured at 4 h intervals until 24 h, or until the gap distance was complete or no longer progressing (Figure 1).

### 2.2. Preparation of Canine Fresh A-PRF and Lyophilized A-PRF

A-PRF preparations were performed according to a previously published protocol by Ghanaati and colleagues [22], with slight modifications. Briefly, immediately after the 10 mL of venous blood was collected in a 10 mL blood-collecting tube without an anticoagulant (BD Vacutainer^®^, Plymouth, UK), the sample was centrifuged at 100 g for 14 min using a benchtop centrifuge (Hettich^®^ EBA 21, Tuttlingen, Germany) at room temperature. After centrifugation, a formed fibrin clot formation in the middle of the tube, between the red corpuscles at the bottom and the acellular plasma at the top, was removed by using sterile forceps, and then was separated from the red corpuscle layer using sterile scissors, with a thin layer of RBC being retained. For lyophilized A-PRF, the collected A-PRF was frozen at −80 °C for 30 min and freeze-dried at −51 °C for 24 h using a lyophilizer (Lyophilization Systems Inc., Kingston, NY USA), as previously described [32,34]. Then, the lyophilized A-PRF samples were stored at −20 °C. Thereafter, fresh A-PRF and lyophilized A-PRF were transferred to 6-well cell culture plates with 1 mL of Dulbecco’s Modified Eagle Medium (DMEM) (Gibco, Life Technologies Corporation, New York, NY, USA) and processed for further investigation, including the release of growth factors and the biological activity on fibroblast proliferation and migration (Figure 1). 

### 2.3. Enzyme-Linked Immunosorbent Assay (ELISA) for the Measurement of Growth Factors

To measure growth factor release, both A-PRF preparations were transferred into a 6-well plate. One milliliter of DMEM (Gibco, Life Technologies Corporation, New York, NY, USA) was added to each sample. The plate was then placed in a 5% CO_2_ incubator at 37 °C to allow for growth factors to be released into the culture media during the three-day period of the study. At 1 h, 24 h, and 72 h, 1 mL of the culture media was collected, kept at −20 °C, and replaced with 1 mL of additional fresh culture media. The collected samples at each time point were analyzed for canine TGF-β1, VEGFA, and PDGF-BB using commercial ELISA kits, including TGF-β1 (MyBioSource, Inc., San Diego, CA, USA), VEGFA (Abcam, Cambridge, UK), and PDGF-BB (Abcam, Cambridge, UK), respectively, according to the manufacturers’ instructions. Optical density was assessed using a microplate reader at 450 nm. The absorbance measured at 630 nm was subtracted from that measured at 450 nm for an optical density correction. The measurement was performed in duplicate.

### 2.4. Evaluation of Biological Properties 

#### 2.4.1. Cell Culture

Swiss 3T3 albino mouse fibroblasts (IFO50417, JCRB cell bank) were cultured in a 5% CO_2_ incubator at 37 °C with 95% humidity in DMEM (Gibco, Life Technologies Corporation, New York, NY, USA), 10% fetal bovine serum (FBS) (Gibco, Life Technologies Limited, Paisley, UK), and 1% antibiotics (Gibco, Life Technologies Limited, Paisley, UK). The media were changed two times per week. All cells were detached from the tissue culture plastic by using 0.25% EDTA-trypsin (CORNING, Mediatech, Inc., Manassas, VA, USA) before reaching confluences. The cells used for the experimental seeding were obtained from passages 11–13.

The conditioned media were prepared according to Li and colleagues [32] and Fujioka and colleagues [24], with slight modifications. Briefly, either fresh or lyophilized A-PRF membranes were placed in 6-well cell culture plates, containing 1 mL of serum-free DMEM (Gibco, Life Technologies Corporation, New York, NY, USA), and incubated in a 5% CO_2_ incubator at 37 °C with 95% humidity for 7 days. As control conditioned media, 1 mL of DMEM without an A-PRF membrane was incubated in the same condition as previously described. All conditioned media were collected every 48 h, and the fresh medium was added to replace the wells after collection. Thereafter, conditioned media were utilized in future experiments at two concentrations, including 5% and 20% of the total volume, as previously described [24,35]. 3T3 fibroblasts were seeded with 5% and 20% conditioned media from fresh A-PRF, lyophilized A-PRF, as well as the control contained within a growth medium at a density of 12,500 cells/mL in a 96-well plate for the cell proliferation experiments [32] and 300,000 cells/mL in 24-well plates for the scratch wound assay [36].

#### 2.4.2. Proliferation Assay 

Fibroblasts of the 3T3 type were seeded into 96-well cell culture plates at a concentration of 12,500 cells/mL and cultured for 48 h. Conditioned media from fresh A-PRF, lyophilized A-PRF, as well as the control, at either 5% or 20% of the total volume, were added to each well. Cell proliferation was quantified using an MTT colorimetric assay (Invitrogen, Thermo Fisher Scientific, Life Technologies Corporation, Eugene, Oregon, USA) at 1, 3, 5, and 7 days according to the manufacturer’s instructions. Briefly, at desired time points, 10 µL of an MTT reagent at a concentration of 5 mg/mL was added and incubated at 37 °C with 95% humidity for another 2 h. Subsequently, the solution part was discarded and 100 µL of dimethyl sulfoxide (DMSO) (Sigma^®^ Life Science, St. Louis, MO, USA) was added to dissolve the dark blue crystals of MTT formazan. The spectrophotometric absorbance (optical density) of the obtained solution was read using a microplate reader at 570 nm. The experiment was performed in triplicate, with two independent experiments performed. Cell proliferation numbers were presented as a percentage, which was calculated using the following formula (Equation (1)):

(1) $$\frac{\mathrm{OD}\;\mathrm{of}\;\mathrm{test}}{\mathrm{OD}\;\mathrm{of}\;\mathrm{control}\;\mathrm{at}\;1\;\mathrm{day}\;}\times 100$$

#### 2.4.3. Scratch Wound Assay 

Fibroblast cells of the 3T3 type were seeded into 24-well cell culture plates at a concentration of 300,000 cells/mL. They were maintained in a 5% CO_2_ incubator at 37 °C with 95% humidity for 24 h to permit cell adhesion and the formation of a confluent monolayer, and then a sterile 200 µL pipette tip was used to create a straight scratch wound, as previously described [37]. After scratching, the cell was gently washed with media to remove the detached cells. The removed media were replaced with 5% and 20% conditioned media of fresh A-PRF and lyophilized A-PRF, as well as of the control. To reduce cell proliferation, a low serum concentration, 0.5% FBS, was used within a growth medium [38]. After scratching, the cell-cultured plates were incubated, and the gap distance was monitored by collecting images at 4 h intervals until 24 h, or until wound closure was complete. All of the scratch assays were performed in triplicate with two independent trials. 

Images were captured with an inverted microscope (Leica DM IL LED, Leica, Germany) and a digital camera (Leica DFC450 C, Leica Microsystems Ltd., Heerbrugg, Switzerland). Then, the images were analyzed using Leica Application Suite V4.12 software to measure the width of the scratch at three points along the vertical axis of the image, equating to the top, middle, and bottom of the field of view. The data have been presented as a percentage of wound closure, which can be calculated using the following formula (Equation (2)):

(2) $$\frac{A0h-Axh}{A0h}x100$$

where *A0h* was the area of the wound measured immediately after scratching (time of zero), and *Axh* was the area of the wound measured *x* hours after scratching. 

### 2.5. Statistical Analysis 

Means and standard errors (SEs) were used to describe the data. Due to the use of data from the same dogs, repeated measure analyses were used to determine differences in the released growth factors, wound healing assays, and cell proliferation between the fresh and lyophilized A-PRF using a generalized linear mixed model (Proc mixed, SAS). For all models, data from the same dogs were treated as a random effect, and the correlation structures were autoregression type 1. All independent variables were treated as categorical variables. For each growth factor release model, the interaction of the group (fresh and lyophilized A-PRF) and the time release (1, 24, and 72 h) was an independent variable, and the adjusted means of the interactions (least square means of the mixed model) were calculated and compared. Comparisons of cell proliferation among the control, fresh A-PRF, and lyophilized A-PRF were separately analyzed on days 1, 3, 5, and 7, respectively. For the scratch wound assays, a percentage of wound closure was defined as a dependent variable, the interaction of the A-PRF groups (control, fresh, and lyophilized) and their percentages (5% and 20%) was an independent variable, and the analyses were separately analyzed for data at 8, 12, 16, 20, and 24 h. The least-square means of the interaction of the mixed model were calculated and compared. The significance level was defined at *p* < 0.05, and the tendency was defined at *p* < 0.10. Different letters among ^a,b,c^ indicated differences between groups within the specified time in all comparisons at *p* < 0.05. For the growth factor release comparisons, different letters among ^i,j^ and ^m,n^ indicate a significant difference between the time points of the fresh A-PRF and the lyophilized A-PRF, respectively. 

## 3. Results 

### 3.1. Growth Factor Release from the Fresh and Lyophilized Canine A-PRF

**Figure 2:**
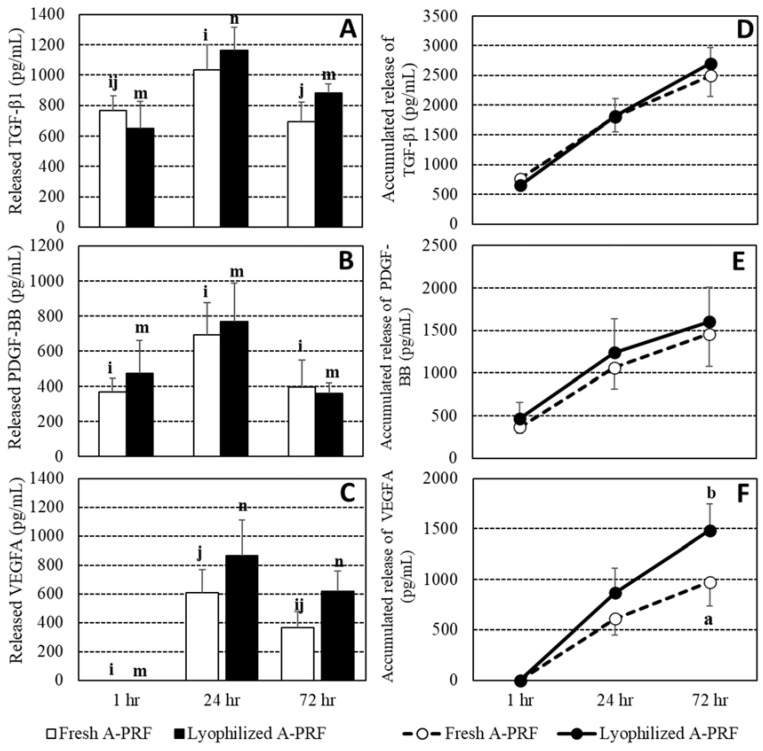
Releases and accumulated releases, as bar and line charts, respectively, of growth factors, including transforming growth factor-beta1 (TGF-β1) (**A**,**D**), platelet-derived growth factor-BB (PDGF-BB) (**B**,**E**), and vascular endothelial growth factor-A (VEGFA) (**C**,**F**), between the fresh and lyophilized advanced platelet-rich fibrin A-PRF at 1 h, 24 h, and 72 h. ^I,j^ and ^m,n^: different letters indicating differences between time points within the fresh A-PRF and the lyophilized A-PRF, respectively. ^a,b^: different letters indicating differences between the fresh and the lyophilized A-PRF at a specified time at *p* < 0.05.

Due to an undesired blood clot for a blood sample from a dog, only data from five dogs were included in the final analysis. The concentration of each growth factor was shown in Tables S1 and S2. Overall the means and their ranges of the TGF-β1, VEGFA, and PDGF-BB releases of fresh A-PRF and lyophilized A-PRF were 2494.4 (1222.2–3357.1) and 2700.0 (2067.9–3628.0), 975.4 (191.4–1610.0) and 1485.4 (821.0–2325.8), and 1457.3 (395.2–2587.2) and 1601 (910.0–3191.7) pg/mL, respectively. The overall VEGFA release of lyophilized A-PRF was higher than that released from fresh A-PRF (*p* < 0.05). The means and SEs of growth factor releases and their accumulated releases in both fresh and lyophilized A-PRF at 1, 24, and 72 h are shown in Figure 2. At 1 h, the VEGFA release was not detected. All growth factor releases were lowest at 1 h and were significantly increased at 24 h, before decreasing at 72 h. In the comparison between the fresh and lyophilized A-PRF, the TGF-β1 of the lyophilized A-PRF had a higher tendency than that of the fresh A-PRF at 72 h (*p* = 0.08), while VEGFA releases at 24 h and 72 h of the lyophilized A-PRF were at the edge of having a higher tendency than that of the fresh A-PRF, at *p* = 0.12 and *p* = 0.12, respectively. For the accumulated release of growth factors, no difference between the fresh and lyophilized A-PRF of TGF-β1 and PDGF-BB was observed, but higher cumulative releases of VEGFA of the lyophilized compared to the fresh A-PRF were found at 24 h (*p* = 0.13) and 72 h (*p* < 0.01). 

### 3.2. Influence of Fresh A-PRF and Lyophilized Canine A-PRF on Cell Proliferation

**Figure 3:**
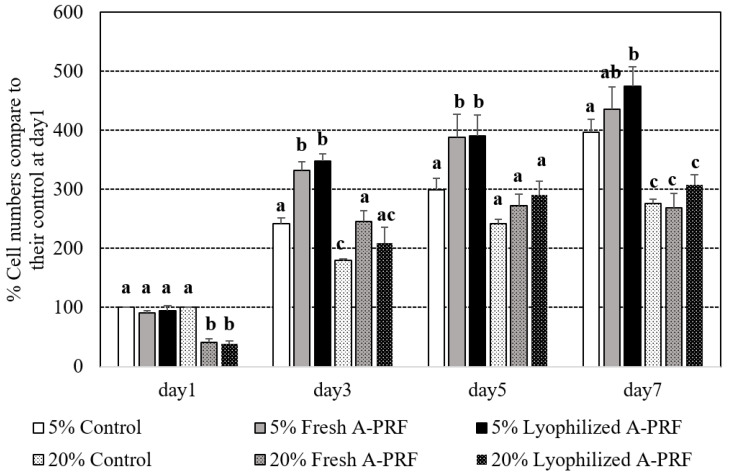
Percentages of different cell proliferation numbers of the standard cultured media supplemented with 5% and 20% of fresh and lyophilized A-PRF compared to the control media of their supplemented percentages at day 1. ^a,b,c^: different letters indicating significant differences among the control and fresh as well as lyophilized A-PRF within the same day.

Percentages of the cell proliferation numbers of the media supplemented with 5% and 20% of the fresh and lyophilized A-PRF compared to the control medium on day 1 of their supplemented percentages are shown in Figure 3. All of the groups spontaneously increased their percentages of cell proliferation during the experimental period. On day 1 after culturing, the proliferation number in the 20% supplementations of both the fresh and lyophilized A-PRF was less than that of the 20% control and all of the 5% groups (*p* < 0.05). From day 3 to the end of the study, the 5% groups of the fresh and lyophilized A-PRF had significantly higher cell proliferations than those of all of the 20% groups. Compared to the 5% control group, the cell proliferation of the 5% fresh A-PRF group was significantly higher on day 3 and day 5, while that of the 5% lyophilized A-PRF group was significantly higher on day 3, day 5, and day 7. No significant differences between fresh and lyophilized A-PRF in all of the percentages in any of the culture periods were observed. 

### 3.3. Influence of Fresh A-PRF and Lyophilized Canine A-PRF on Wound Closure

**Figure 4:**
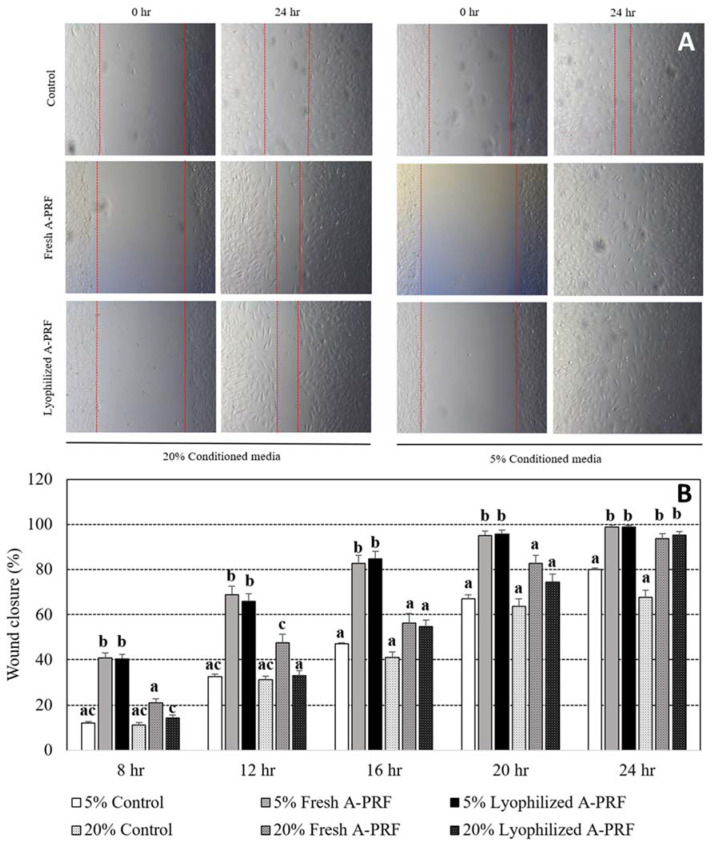
Examples of the representative time lapse images of the fibroblast wound healing assay at 0 and 24 h for each group (**A**). Percentages of wound closure after culturing for 8, 12, 16, 20, and 24 h in the standard cultured medium supplemented with 5% and 20% of the control media, the fresh A-PRF, and the lyophilized A-PRF (**B**). ^a,b,c^: different letters indicating significant differences between the control, fresh A-PRF, and lyophilized A-PRF within the same specified time.

Representative time lapse images of fibroblast scratch wound assays immediately and 24 h after wound scratching among the groups are shown in Figure 4A. The straight lines in each figure indicate the area of the fibroblast cells that moved to heal the wound, and the gap distance between the two lines was used to determine wound closure percentages. No gap, which indicated complete wound healing, was observed in 5% fresh A-PRF and 5% lyophilized A-PRF at 24 h, as shown in Figure 4A. The percentages of wound closure from 5% and 20% cultured conditions of the control and fresh as well as lyophilized A-PRF at 4 h intervals, until 24 h, are shown in Figure 4B. The percentages of wound closure in all of the groups were increased with the increased culture times, in which the averaged wound closure of 5% fresh and 5% lyophilized A-PRF at 24 h was close to 100%. Both 5% fresh A-PRF and 5% lyophilized A-PRF had higher percentages of wound closure than the 5% control and all of the 20% groups in almost all of the culture times (*p* < 0.05), except at 24 h, in which these 5% A-PRF treatments had higher percentages of wound closure than the 5% control group and the 20% control group. No difference between fresh and lyophilized A-PRF was observed within the 5% and 20% cultured conditions at all times, except at 8 h and 12 h, in which a higher percentage of wound closure was observed from 20% fresh A-PRF than 20% lyophilized A-PRF. 

## 4. Discussion

Although fresh PRF products have been encouraged to be used in soft tissue and hard tissue regeneration for both human and veterinary medicine [9,25,26,39], the application of PRF remains reliant upon a fresh preparation due to the short half-life of the product. Moreover, the A-PRF produced more growth factors [23], cell migration, and proliferation of gingival fibroblasts than the traditional PRF [24]. Therefore, a lyophilized preparation has been developed to improve the stability and storage potential [30,31], and this study has been the first one to evaluate the lyophilized A-PRF from canine blood samples, which must be directly beneficial to veterinary medicine. TGF-β1, PDGF, and VEGF are the three main growth factors presented in PRF products, and are produced by platelets and leukocytes [23]. These growth factors play an important role in wound healing, which is composed of 4 overlapping phases: hemostasis, inflammation, proliferation, and remodeling [40]. Growth factors recruit and stimulate the cells, such as endothelial cells, fibroblasts, and keratinocytes, to heal wounds, i.e., the proliferation phase, as in the scratch wound closure assay, and then the fibroblasts deposit new extracellular matrix proteins to form granulation tissue, as in the cell proliferation assay (takes ~7 days) [41]. Regardless of the fresh and lyophilized A-PRF, this study found that TGF-β1 and PDGF-BB started to be released at 1 h, while VEGFA started to be released at 24 h (Figure 2). One of the hypothesized reasons for this is the fact that, while TGF-β1 and PDGF act early in the inflammation and proliferation phases, VEGF is fundamental in the proliferative phase [42]. The maximum releases of growth factors from both fresh and lyophilized A-PRF were at 24 h, and were subsequently decreased. These data shared a similar pattern of PDGF release with a previous study from Ngah and colleagues [43]. 

PRF is an important reservoir for growth factors since they release a high concentration of biologically active proteins that support the recruitment of cells from surrounding host tissue [17]. Regardless of the preparation, the present study demonstrated that canine A-PRF released TGF-β1, PDGF-BB, and VEGFA. The activities of these growth factors were seen in the results of the scratch wound assays, in which fibroblast cultures supplemented with any A-PRF had faster wound closure than the control at 24 h (Figure 4B). The mechanism of A-PRF action that promotes mouse fibroblast migration might be mediated by the release of growth factors. At an early stage, fibroblast proliferation and chemotaxis to the wound site were stimulated by PDGF and TGF-β1 [4]. The chemotactic effect of growth factors, including PDGF and TGF-β1, contributes to the cell migration of fibroblasts during wound healing [4]. Furthermore, PDGF coordinates with collagen matrices to increase the expression of fibroblast α2, α3, and α5 integrins, which are required for enhanced migration [44]. Although the cell proliferation in all of the groups (especially in 5% conditioned media) was comparable at 24 hr, the increase in cell proliferation from 5% fresh and lyophilized A-PRF was higher than that of the control starting at day 3 to day 5 and day 7 for 5% lyophilized A-PRF (Figure 3). 

Regarding the different percentages of cell culture conditions, the 5% lyophilized A-PRF and 5% fresh A-PRF showed significantly higher wound closure percentages and cell proliferation percentages than those of the 20% cultured condition. This finding was supported by a previous study by Steller and colleagues [35], in which 5% supplementation was better than 20% supplementation. Oshima and colleagues [45,46] revealed that high TGF- β1 and VEGF concentration might contribute to tissue degradation and might relate to the lower cell proliferation and wound healing capacity of 20% compared to that of 5% conditioned media. 

Comparisons of the wound healing capacity between the fresh and the lyophilized A-PRF were performed throughout the study. Most assays could not find different results between both A-PRF supplementation cultures. This study demonstrated that the lyophilization process did not deteriorate the release of growth factors in vitro, wound closure, and cell proliferation. Based on this finding, it became clear that the biological function of growth factors could be preserved by using the lyophilization process. A previous study also indicated that the lyophilized platelet preparation of equine blood could stimulate fibroblast migration and proliferation [47]. The releases of growth factors from canine lyophilized A-PRF in 72 h might indicate that lyophilization also maintained the three-dimensional structure of fibrin, causing the continuing release of the growth factor, as the fresh form did. Although the released growth factors were not measured after 72 h, this study found that the cell proliferation of the lyophilized A-PRF group was higher than that of the control group from 72 h to 168 h. This indicated that the lyophilized A-PRF might have continued to release growth factors during a higher cell proliferation percentage. Moreover, the existence of a three-dimensional fibrin network in the PRF products could reduce the susceptibility of growth factors to proteolytic degradations but increased fibroblast proliferation and migration [17,48,49]. The TGF-β1 and VEGFA release of lyophilized A-PRF showed a tendency higher than that of fresh A-PRF (Figure 2), and the significantly higher accumulated release of VEGFA coincided with the increased percentage of cell proliferation of lyophilized A-PRF over the fresh variant, but not significantly. These results were similar to a previous study that showed that the lyophilized process improved the release of growth factors from lyophilized PRF [32]. 

## 5. Conclusions

The present study demonstrated that both formulations of canine A-PRF matrices, including fresh and lyophilized forms, were able to release various growth factors that promote wound regeneration. Interestingly, lyophilized canine A-PRF demonstrated the tendency of larger releases of certain growth factors, including the significantly higher accumulated release of VEGFA. Based on these findings, it became clear that the lyophilization process can preserve growth factor release as well as the biological activity of canine A-PRF matrices, in addition to encouraging the use of canine lyophilized A-PRF as a biological material for promoting wound healing. As mentioned above, a variety of therapeutic materials for example nanomedicine, hydrogel, natural polysaccharides, and phenolic compounds and proteins have been developed in parallel with the uses of platelets for improvement of wound healing. Future investigations need to be undertaken to determine advantages and disadvantages among these recent techniques. 

## Data Availability

Not applicable.

## Supplementary Materials

The following supporting information can be downloaded at: https://www.mdpi.com/article/10.3390/vetsci9100566/s1, Table S1: Growth factors released from Canine lyophilized A-PRF, Table S2: Growth factors released from Canine fresh A-PRF.

## References

[1] Animal Wound Care Market Size, Share & Trends Analysis Report By Product (Surgical, Advanced), By Animal Type (Livestock, Companion), By End User, By Distribution Channel, By Region, And Segment Forecasts, 2021–2028. https://www.grandviewresearch.com/industry-analysis/animal-wound-care-market.

[2] Swaim S.F., Henderson R. (1990). Small Animal Wound Management.

[3] Boulton A.J., Vileikyte L., Ragnarson-Tennvall G., Apelqvist J. (2005). The global burden of diabetic foot disease. Lancet.

[4] Lorenz H.P., Longaker M.T., Norton J.A., Barie P.S., Bollinger R.R., Chang A.E., Lowry S.F., Mulvihill S.J., Pass H.I., Thompson R.W. (2008). Wounds: Biology, Pathology, and Management. Surgery: Basic Science and Clinical Evidence.

[5] Morton L.M., Phillips T.J. (2016). Wound healing and treating wounds: Differential diagnosis and evaluation of chronic wounds. J. Am. Acad. Dermatol..

[6] Xian C., Zhang Z., You X., Fang Y., Wu J. (2022). Nanosized Fat Emulsion Injection Modulating Local Microenvironment Promotes Angiogenesis in Chronic Wound Healing. Adv. Funct. Mater..

[7] Xu Z., Liu G., Li Q., Wu J. (2022). A novel hydrogel with glucose-responsive hyperglycemia regulation and antioxidant activity for enhanced diabetic wound repair. Nano Res..

[8] Su X., Xian C., Gao M., Liu G., Wu J. (2021). Edible Materials in Tissue Regeneration. Macromol. Biosci..

[9] Miron R.J., Fujioka-Kobayashi M., Bishara M., Zhang Y., Hernandez M., Choukroun J. (2017). Platelet-Rich Fibrin and Soft Tissue Wound Healing: A Systematic Review. Tissue Eng. Part Rev..

[10] Rozman P., Bolta Z. (2007). Use of platelet growth factors in treating wounds and soft-tissue injuries. Acta Derm. Alp. Pannonica. Adriat..

[11] Carter M.J., Fylling C.P., Parnell L.K. (2011). Use of platelet rich plasma gel on wound healing: A systematic review and meta-analysis. Eplasty.

[12] Oneto P., Zubiry P.R., Schattner M., Etulain J. (2020). Anticoagulants Interfere with the Angiogenic and Regenerative Responses Mediated by Platelets. Front. Bioeng. Biotechnol..

[13] Dohan Ehrenfest D.M., Rasmusson L., Albrektsson T. (2009). Classification of platelet concentrates: From pure platelet-rich plasma (P-PRP) to leucocyte- and platelet-rich fibrin (L-PRF). Trends Biotechnol..

[14] Choukroun J., Adda F., Schoefer C., Vervelle A. (2000). Uneopportunité enparo-implantologie: Le PRF. Implantodontie.

[15] Dohan D.M., Choukroun J., Diss A., Dohan S.L., Dohan A.J.J., Mouhyi J., Gogly B. (2006). Platelet-rich fibrin (PRF): A second-generation platelet concentrate. Part I: Technological concepts and evolution. Oral Surg. Oral Med. Oral Pathol. Oral Radiol. Endodontol..

[16] Choukroun J., Diss A., Simonpieri A., Girard M.O., Schoeffler C., Dohan S.L., Dohan A.J., Mouhyi J., Dohan D.M. (2006). Platelet-rich fibrin (PRF): A second-generation platelet concentrate. Part IV: Clinical effects on tissue healing. Oral Surg. Oral Med. Oral Pathol. Oral Radiol. Endodontol..

[17] Dohan D.M., Choukroun J., Diss A., Dohan S.L., Dohan A.J., Mouhyi J., Gogly B. (2006). Platelet-rich fibrin (PRF): A second-generation platelet concentrate. Part II: Platelet-related biologic features. Oral Surg. Oral Med. Oral Pathol. Oral Radiol. Endodontol..

[18] Dohan D.M., Choukroun J., Diss A., Dohan S.L., Dohan A.J.J., Mouhyi J., Gogly B. (2006). Platelet-rich fibrin (PRF): A second-generation platelet concentrate. Part III: Leucocyte activation: A new feature for platelet concentrates?. Oral Surg. Oral Med. Oral Pathol. Oral Radiol. Endodontol..

[19] Vahabi S., Vaziri S., Torshabi M., Rezaei Esfahrood Z. (2015). Effects of Plasma Rich in Growth Factors and Platelet-Rich Fibrin on Proliferation and Viability of Human Gingival Fibroblasts. J. Dent..

[20] Clipet F., Tricot S., Alno N., Massot M., Solhi H., Cathelineau G., Perez F., De Mello G., Pellen-Mussi P. (2012). In vitro effects of Choukroun’s platelet-rich fibrin conditioned medium on 3 different cell lines implicated in dental implantology. Implant. Dent..

[21] Hartshorne J., Gluckman H. (2016). A comprehensive clinical review of Platelet Rich Fibrin (PRF) and its role in promoting tissue healing and regeneration in dentistry. Int. Dent. Afr. Ed..

[22] Ghanaati S., Booms P., Orlowska A., Kubesch A., Lorenz J., Rutkowski J., Landes C., Sader R., Kirkpatrick C., Choukroun J. (2014). Advanced platelet-rich fibrin: A new concept for cell-based tissue engineering by means of inflammatory cells. J. Oral. Implantol..

[23] Kobayashi E., Flückiger L., Fujioka-Kobayashi M., Sawada K., Sculean A., Schaller B., Miron R.J. (2016). Comparative release of growth factors from PRP, PRF, and advanced-PRF. Clin. Oral. Investig..

[24] Fujioka-Kobayashi M., Miron R.J., Hernandez M., Kandalam U., Zhang Y., Choukroun J. (2017). Optimized Platelet-Rich Fibrin with the Low-Speed Concept: Growth Factor Release, Biocompatibility, and Cellular Response. J. Periodontol..

[25] Hamed M.A., Abouelnasr K.S., El-Adl M., Abo Elfadl E.A., Farag A., Lashen S. (2019). Effectiveness of Allogeneic Platelet-Rich Fibrin on Second-Intention Wound Healing of Experimental Skin Defect in Distal Limb in Donkeys (Equus asinus). J. Equine Vet. Sci..

[26] Soares C.S., Barros L.C., Saraiva V., Gomez-Florit M., Babo P.S., Dias I.R., Reis R.L., Carvalho P.P., Gomes M.E. (2018). Bioengineered surgical repair of a chronic oronasal fistula in a cat using autologous platelet-rich fibrin and bone marrow with a tailored 3D printed implant. J. Feline Med. Surg..

[27] Soares C.S., Dias I.R., Pires M.A., Carvalho P.P. (2021). Canine-Origin Platelet-Rich Fibrin as an Effective Biomaterial for Wound Healing in Domestic Cats: A Preliminary Study. Vet. Sci..

[28] Soares C.S., Babo P.S., Faria S., Pires M.A., Carvalho P.P. (2021). Standardized Platelet-Rich Fibrin (PRF) from canine and feline origin: An analysis on its secretome pattern and architectural structure. Cytokine.

[29] Davis V.L., Abukabda A.B., Radio N.M., Witt-Enderby P.A., Clafshenkel W.P., Cairone J.V., Rutkowski J.L. (2014). Platelet-rich preparations to improve healing. Part I: Workable options for every size practice. J. Oral. Implantol..

[30] Haugh M.G., Murphy C.M., O’Brien F.J. (2010). Novel freeze-drying methods to produce a range of collagen-glycosaminoglycan scaffolds with tailored mean pore sizes. Tissue Eng. Part Methods.

[31] Choi C.W., Kim B.S., Seo J.H., Shin S.W., Kim Y.H., Kim J.S. (2001). Long-term engraftment stability of peripheral blood stem cells cryopreserved using the dump-freezing method in a -80 degrees C mechanical freezer with 10% dimethyl sulfoxide. Int. J. Hematol..

[32] Li Q., Reed D.A., Min L., Gopinathan G., Li S., Dangaria S.J., Li L., Geng Y., Galang M.T., Gajendrareddy P. (2014). Lyophilized platelet-rich fibrin (PRF) promotes craniofacial bone regeneration through Runx2. Int. J. Mol. Sci..

[33] Xu F., Zou D., Dai T., Xu H., An R., Liu Y., Liu B. (2018). Effects of incorporation of granule-lyophilised platelet-rich fibrin into polyvinyl alcohol hydrogel on wound healing. Sci. Rep..

[34] Kardos D., Hornyák I., Simon M., Hinsenkamp A., Marschall B., Várdai R., Kállay-Menyhárd A., Pinke B., Mészáros L., Kuten O. (2018). Biological and Mechanical Properties of Platelet-Rich Fibrin Membranes after Thermal Manipulation and Preparation in a Single-Syringe Closed System. Int. J. Mol. Sci..

[35] Steller D., Herbst N., Pries R., Juhl D., Hakim S.G. (2019). Positive impact of Platelet-rich plasma and Platelet-rich fibrin on viability, migration and proliferation of osteoblasts and fibroblasts treated with zoledronic acid. Sci. Rep..

[36] Fronza M., Heinzmann B., Hamburger M., Laufer S., Merfort I. (2009). Determination of the wound healing effect of Calendula extracts using the scratch assay with 3T3 fibroblasts. J. Ethnopharmacol..

[37] Martinotti S., Ranzato E. (2020). Scratch Wound Healing Assay. Methods Mol. Biol..

[38] Grada A., Otero-Vinas M., Prieto-Castrillo F., Obagi Z., Falanga V. (2017). Research Techniques Made Simple: Analysis of Collective Cell Migration Using the Wound Healing Assay. J. Investig. Dermatol..

[39] Barbon S., Stocco E., Macchi V., Contran M., Grandi F., Borean A., Parnigotto P.P., Porzionato A., De Caro R. (2019). Platelet-Rich Fibrin Scaffolds for Cartilage and Tendon Regenerative Medicine: From Bench to Bedside. Int. J. Mol. Sci..

[40] Barrientos S., Stojadinovic O., Golinko M.S., Brem H., Tomic-Canic M. (2008). Growth factors and cytokines in wound healing. Wound Repair Regen..

[41] Clark R.A.F., Clark R.A.F., Henson P.M. (1988). Overview and General Considerations of Wound Repair. The Molecular and Cellular Biology of Wound Repair.

[42] Bao P., Kodra A., Tomic-Canic M., Golinko M.S., Ehrlich H.P., Brem H. (2009). The role of vascular endothelial growth factor in wound healing. J. Surg. Res..

[43] Ngah N.A., Dias G.J., Tong D.C., Mohd Noor S.N.F., Ratnayake J., Cooper P.R., Hussaini H.M. (2021). Lyophilised Platelet-Rich Fibrin: Physical and Biological Characterisation. Molecules.

[44] Xu J., Clark R.A. (1996). Extracellular matrix alters PDGF regulation of fibroblast integrins. J. Cell. Biol..

[45] Ohshima M., Yamaguchi Y., Ambe K., Horie M., Saito A., Nagase T., Nakashima K., Ohki H., Kawai T., Abiko Y. (2016). Fibroblast VEGF-receptor 1 expression as molecular target in periodontitis. J. Clin. Periodontol..

[46] Ohshima M., Yamaguchi Y., Matsumoto N., Micke P., Takenouchi Y., Nishida T., Kato M., Komiyama K., Abiko Y., Ito K. (2010). TGF-β signaling in gingival fibroblast-epithelial interaction. J. Dent. Res..

[47] Tablin F., Walker N.J., Hogle S.E., Pratt S.M., Norris J.W. (2008). Assessment of platelet growth factors in supernatants from rehydrated freeze-dried equine platelets and their effects on fibroblasts in vitro. Am. J. Vet. Res..

[48] Rybarczyk B.J., Lawrence S.O., Simpson-Haidaris P.J. (2003). Matrix-fibrinogen enhances wound closure by increasing both cell proliferation and migration. Blood.

[49] Sahni A., Baker C.A., Sporn L.A., Francis C.W. (2000). Fibrinogen and fibrin protect fibroblast growth factor-2 from proteolytic degradation. Thromb. Haemost..

