# Supplementary figures and images for: In Vitro Assessment of Lyophilized Advanced Platelet-Rich Fibrin from Dogs in Promotion of Growth Factor Release and Wound Healing
Source: Vet Sci. 2022 Oct 15;9(10):566. doi: 10.3390/vetsci9100566 (PMC9610920; doi:10.3390/vetsci9100566)

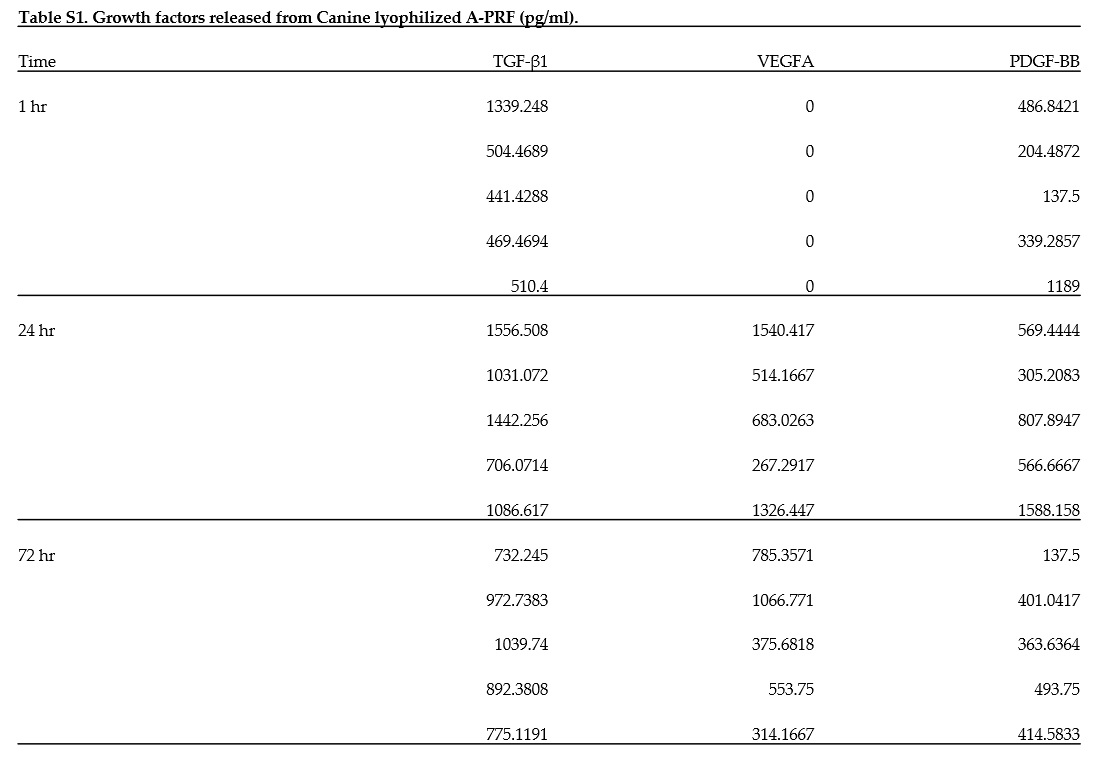

Supplement: Supplementary file 1 [file vetsci-09-00566-s001.zip › Table S1.jpg]

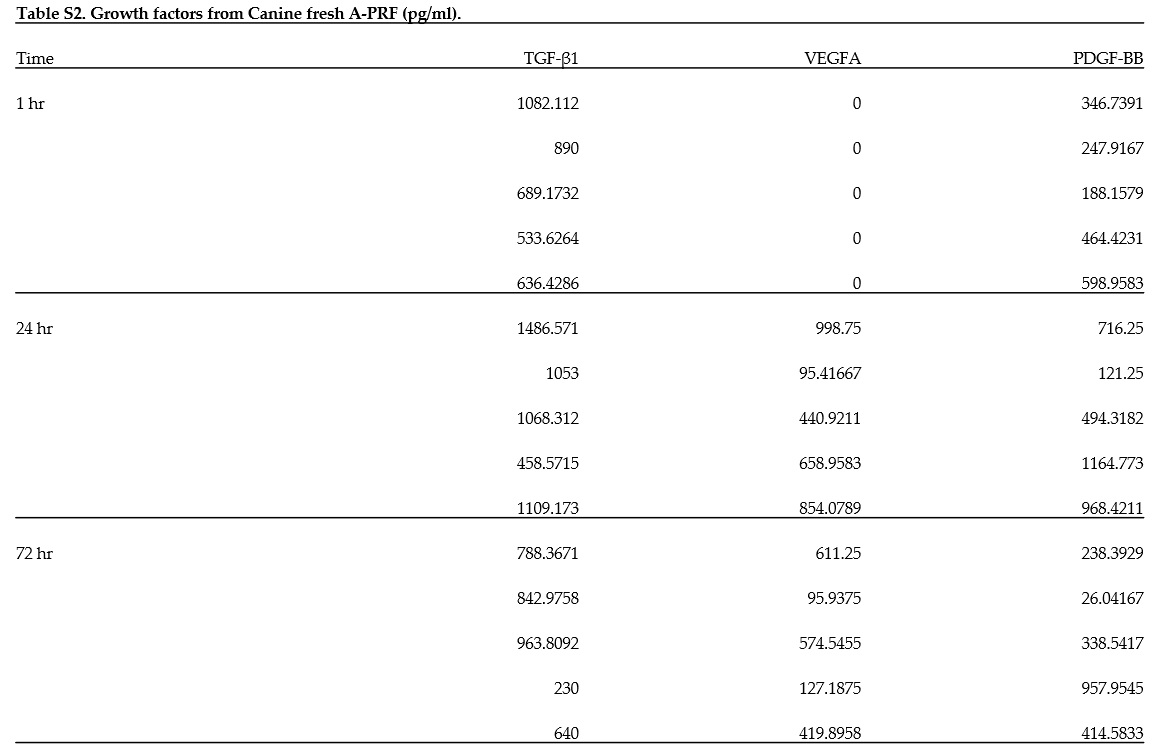

Supplement: Supplementary file 1 [file vetsci-09-00566-s001.zip › Table S2.jpg]
